# Interactions of dietary nutrient density with genotype and sex in broiler chickens: implications for growth, carcass quality, and nutrient utilization

**DOI:** 10.1016/j.psj.2025.106147

**Published:** 2025-11-21

**Authors:** Carria Xie, Shemil Macelline, Sonia Liu, Mehdi Toghyani

**Affiliations:** a School of Life and Environmental Sciences, Faculty of Science, The University of Sydney, Sydney, NSW 2006, Australia; b Poultry Research Foundation, The University of Sydney, Camden, NSW 2570, Australia

**Keywords:** Broiler genotype, Nutrient density, Nutrient digestibility, Feed efficiency

## Abstract

This study aimed to assess the interactive effects of dietary nutrient density, broiler genotype, and sex on growth performance, breast meat yield and quality, nutrient digestibility and utilization in straight run broilers. A total of 1080 straight-run Ross 308 and 1080 straight-run Cobb 500 chicks were offered 3 dietary treatments formulated to either Ross 308, Cobb 500, or AgriFutures (AGF) nutrients specifications. AGF specifications reduced ME by 100 and 75 kcal/kg in starter and grower diets, and increased ME by 25 kcal/kg in withdrawal compared with Ross 308 guidelines. Each treatment was replicated 8 times with 45 birds per replicate, and fed for starter (0–10 d), grower (10–24 d), finisher (24–35 d), and withdrawal (35–42 d) phases. On day 35, six birds per pen (3 males, 3 females) were euthanized for digesta collection to determine nutrient digestibility; on day 42, a further six birds per pen (3 males, 3 females) were euthanized for carcass yield and breast meat quality assessments. Final BW on day 42 was not affected by nutrient density, however, as a main effect, Cobb birds exhibited faster growth rate and achieved higher BW but also had a higher FCR than Ross (*P* < 0.01). In addition, as a main effect Cobb specification resulted in higher FI and FCR (*P* < 0.01). Despite the higher ME and digestible lysine intake and higher nutrient conversion ratio in Cobb birds (*P* < 0.01), there were no significant differences in nutrient digestibility between the two genotypes (*P* > 0.05). Sex significantly influenced weight gain with female birds exhibiting lower BW and higher fat deposition (*P* < 0.01). Female birds also had lower digestibility of protein, energy and fat compared to males (*P* < 0.01). The present study showed that AGF specifications with reduced dietary ME by 100 and 75 kcal/kg in the starter and grower diets respectively, does not compromise growth performance in straight-run broiler chicks. Cobb broilers responded positively to increased dietary amino acid density (Ross and AGF specifications), which was reflected in lower FCR.

## Introduction

Broiler farming has the lowest greenhouse gas emissions (CO₂-equivalent) per kilogram of edible meat compared to other major livestock systems such as beef, lamb, and pork (Clune et al., 2017). In addition, due to its affordability and lack of major ethical or religious barriers, chicken meat is the most consumed animal protein worldwide (Ravindran, 2024). Feed is not only the largest input cost in broiler farming, but also the biggest contributor to greenhouse gas emissions, both through the sourcing of raw materials and its influence on bird performance (Pelletier, 2008). Dietary energy and protein/amino acids (AA) are essential nutrients and represent the highest cost in broiler diets. Both their density and the balance between energy and AA affect birds’ performance in terms of growth rate, feed efficiency and overall cost-effectiveness of chicken meat production. In response, major broiler breeder companies continually update their recommendations for these key nutrients, focusing on both their optimal levels and the ideal balance, particularly the AA profile relative to energy. However, the nutritional guidelines provided by major broiler breeder companies, notably for Ross 308 and Cobb 500 (Aviagen, 2022a; Cobb Vantress, 2022) vary reflecting the differences in genetic potential and growth trajectories between these two broiler genotypes.

Our findings from the three preceding feeding trials indicated that dietary metabolizable energy (ME) can safely be reduced by 100 kcal/kg in the starter and 75 kcal/kg in the grower diets, compared to current breeder recommendations (Aviagen, 2022a) without negatively impacting growth or feed efficiency (Toghyani et al., 2024; Wang et al., 2025; Xie et al., 2024). These results showed that dietary ME reduction has the potential to lower feed costs without compromising productivity. Although reducing dietary ME in older birds (>24 days) did not affect growth rate, it led to increased feed intake and consequently a higher FCR, with every 50 kcal/kg ME reduction resulting in a 2-point increase in FCR (Toghyani et al., 2024). In the same study, increasing dietary AA density improved growth rate and feed efficiency, independent of ME density, although the magnitude of the response was greater in higher ME diets. However, increasing dietary ME by 50 kcal/kg above breeder recommendations in older birds did not translate into any measurable improvement in performance (Xie et al., 2024). These findings further highlight the responsiveness of modern broiler chickens to nutrient density and how balancing performance gains with economic returns could affect both zootechnical performance and overall feed cost efficiency.

All three previous trials were conducted using off-sex male Ross 308 broiler chickens. In addition to sex, genotype specific traits such as growth trajectory, metabolic rates, and genetic potential may lead to different outcomes in performance and carcass yield component in response to dietary nutrient density (Dozier and Gehring, 2014; Maynard et al., 2023; Tona et al., 2010; England et al., 2023). Likewise, male and females often differ in growth rate, nutrient requirements, and feed efficiency, highlighting the importance of considering both factors in nutritional research (Sakomura et al., 2005; England et al., 2023; Ravindran et al., 2004). While Ross 308 has been extensively studies, there is a notable lack of research involving Cobb 500, especially under Australian production system conditions. Given the physiological and growth pattern differences between these two commercially significant genotypes, understanding their specific responses to dietary nutrient density, particularly ME density and their ratio to AA is essential for designing diets with optimized nutrient density or cost efficiency. Previous studies have demonstrated that a reduction in ME during early feeding phases does not adversely affect the growth performance of Ross 308 birds (Toghyani et al., 2024; Xie et al., 2024). However, Cobb breeder guidelines generally target lower dietary ME, and the line is typically selected on diets based on maize, which has intrinsically higher energy content compared to wheat (Pirgozliev et al., 2015). Accordingly, Cobb birds may exhibit genotype-specific responses to dietary energy manipulation, particularly in wheat-based diets.

Therefore, the current study was designed to investigate the interactive effects of dietary nutrient density, both ME and AA, and broiler genotype on the growth performance and carcass characteristics of straight-run broiler chicks (Ross 308 and Cobb 500) reared to 42 days of age. The study also investigated sex-specific responses in terms of nutrient digestibility, carcass yield components, and breast meat quality.

## Materials and methods

All experimental protocols and procedures for the present study were reviewed and approved by the University of Sydney Animal Ethic Committee (Project number 2022/2185).

### Experimental design and bird management

The feeding study consisted of 3 dietary nutrient densities and 2 genotypes designed as a 3 × 2 factorial arrangement of treatments, three dietary nutrient-density specifications (Ross 308, Cobb 500, or AgriFutures) and 2 genotypes of birds (Ross 308 or Cobb 500). The diets were formulated either to Ross 308 (Aviagen, 2022a), Cobb 500 (Cobb Vantress, 2022), or AgriFutures (AGF) nutrients specification. AGF nutrient specification included a reduction of 100 and 75 kcal/kg of ME in starter and grower diets, and an increase of 25 kcal/kg ME in withdrawal diets compared to Ross 308 specs, and the amino acid density followed Ross 308 recommendations. The AGF finisher diet was identical to the Ross 308 specifications.

Each treatment was replicated 8 times with 45 birds per replicate. A total of 2,160 day-old, straight-run broiler chicks (1,080 Ross 308 and 1,080 Cobb 500) were sourced from a commercial hatchery (Picton, NSW 2571, Australia). The parents-flock ages at hatch were 36 weeks for Cobb and 34 weeks for Ross. Upon arrival, birds were not sexed, and the sex ratio was not controlled at placement. On placement day, birds were group-weighed and allocated to 48 floor pens according to body weight. Initial pen body weight did not differ between the pens for each genotypes (Ross: 1671.5 ± 5.0 g/pen; Cobb: 1714.7 ± 5.0 g/pen).The floor pens measured 1.5 m in width and 3.0 m in depth, with fresh wood shavings used as the litter material to a depth of approximately 3 cm. Water and feed were offered *ad lib* from 0 to 42 days post-hatch. The initial room temperature was set to 33 ± 1.5°C and was maintained for the first week before gradually decreasing to 22 °C by day 20 and maintained until the end of the study. Birds received continuous lighting for the first 24 h, which gradually decreased to 16 h by day 35.

### Diet preparation

Prior to diet formulation, representative subsamples of wheat, soybean meal, meat and bone meal, canola meal and canola seed were analyzed by near-infrared spectroscopy to predict proximate analysis, digestible amino acid concentrations, and ME using AMINONIR®PROX, AMINONIR®NIR, and AMINONIR® NRG (Evonik Nutrition & Care, Hanua, DE), respectively.

Diets were based on wheat (10.5 % CP; ME 3180 kcal/kg), soybean meal (46.5 % CP; ME 2400 kcal/kg), meat and bone meal (47.0 % CP; ME 2000 kcal/kg), solvent canola meal (37.5 % CP; ME 1980 kcal/kg) and canola seed (20.5 % CP; ME 4500 kcal/kg), without any inorganic phosphate sources. A phytase dose of 2000 FYT/kg (Ronozyme HiPhorius 10) was included across all the diets with a matrix of 0.20 % Ca, 0.18 % available P, 0.02 % Na, 30 kcal/kg ME uplift and 50 % of the manufacturer recommended matrix for amino acids. The diets were balanced for the essential amino acids including Lys, Met + Cys, Thr, Trp, Arg, Ile and Val by applying the ideal amino acid ratios recommended by breeders (Aviagen, 2022a; Cobb Vantress, 2022). There was neither a cap nor a minimum set for dietary crude protein. Realtime ingredients pricing were used to formulate the diets and supplemental amino acids including Lys, Met, Thr, Arg, Ile and Val were offered to the diets at market prices (Table 1, Table 2).

**Table 1:** Ingredient composition and calculated nutrient profile of the starter and grower diets.

Ingredients %	Starter 0–10 d			Grower 10–24 d		
	AGF^a^	Ross	Cobb	AGF	Ross	Cobb
Wheat 10.5 %	60.03	58.44	61.16	64.42	64.65	66.80
Soybean meal 46.5 %	25.85	25.40	24.65	20.25	18.40	18.75
Canola meal 36.5 %	6.00	6.00	6.00	6.00	6.00	7.00
Meat and bone meal 47 %	3.25	3.30	3.30	1.20	1.30	1.25
Canola seed	2.00	2.00	2.00	5.00	5.00	3.11
Limestone	0.59	0.58	0.58	0.82	0.79	0.81
Canola oil	0.50	2.40	0.70	0.50	1.80	0.50
Lysine HCL	0.395	0.415	0.355	0.395	0.455	0.415
DL-Methionine	0.340	0.350	0.290	0.295	0.315	0.265
Na Bicarbonate	0.300	0.305	0.280	0.350	0.375	0.360
Vitamin/Mineral Premix^b^	0.200	0.200	0.200	0.200	0.200	0.200
L-Threonine	0.165	0.180	0.155	0.145	0.175	0.150
Sodium Chloride	0.145	0.140	0.155	0.135	0.120	0.130
Choline Chloride	0.080	0.080	0.080	0.070	0.070	0.070
L-Valine	0.054	0.069	0.027	0.053	0.088	0.047
L-Arginine	0.034	0.055	0.027	0.086	0.141	0.101
L-Isoleucine	0.031	0.045	-	0.039	0.073	-
Xylanase	0.010	0.010	0.010	0.010	0.010	0.010
Phytase (2000 FYT/kg)	0.020	0.020	0.020	0.020	0.020	0.020
Cost AUD/ton	$652.9	$678.4	$642.5	$628.4	$646.9	$612.7
Calculated composition						
ME kcal/kg	2875	2975	2900	2975	3050	2950
Crude protein %	23.98	23.66	23.48	21.38	20.69	20.96
Dig.Lys %	1.32	1.32	1.26	1.18	1.18	1.16
Dig.Met %	0.64	0.65	0.59	0.57	0.59	0.54
Dig.Met + Cys %	1.00	1.00	0.95	0.92	0.92	0.88
Dig.Thr %	0.88	0.88	0.86	0.79	0.79	0.78
Dig.Ile %	0.88	0.88	0.84	0.80	0.80	0.74
Dig.Leu %	1.49	1.46	1.46	1.34	1.28	1.31
Dig.Trp %	0.26	0.25	0.25	0.23	0.22	0.23
Dig.Arg %	1.40	1.40	1.36	1.27	1.27	1.25
Dig.His %	0.51	0.50	0.50	0.46	0.44	0.45
Dig.Val %	1.00	1.00	0.96	0.91	0.91	0.88
Crude Fat %	3.11	4.94	3.32	4.19	5.47	3.48
Crude Fibre %	3.38	3.32	3.36	3.67	3.60	3.49
Starch %	39.0	38.0	39.7	41.8	41.9	43.3
Ca %	0.95	0.95	0.95	0.80	0.80	0.80
Available P %	0.50	0.50	0.50	0.40	0.40	0.40
Total P %	0.59	0.58	0.59	0.46	0.46	0.46
Na %	0.20	0.20	0.20	0.20	0.20	0.20
Cl %	0.24	0.24	0.24	0.23	0.23	0.23
K %	0.82	0.80	0.80	0.75	0.72	0.73
DEB^c^ mEq/kg	228	225	224	215	206	209

a AGF: AgriFutures specifications.

b The premix contained (per kg of diet): [IU] retinol, 12,000; cholecalciferol, 5,000; [mg] tocopherol, 50; menadione, 3; thiamine, 3; riboflavin, 9; pyridoxine, 5; cobalamin, 0.025; niacin, 50; pantothenate, 10; folate, 2; biotin, 0.2; copper, 20; iron, 40; manganese, 110; cobalt, 0.25; iodine, 1; molybdenum, 2; zinc, 90; selenium, 0.3.

c Dietary electrolyte balance.

**Table 2:** Ingredient composition and calculated nutrient profile of the finisher and withdrawal diets.

Ingredients %	Finisher 24–35 d			Withdrawal 35–42 d		
	AGF^a^	Ross	Cobb	AGF	Ross	Cobb
Wheat 10.5 %	64.16	64.16	66.52	68.30	69.20	72.00
Soybean meal 46.5 %	15.10	15.10	13.95	12.40	12.05	10.35
Canola meal 36.5 %	6.00	6.00	6.00	7.00	7.00	7.00
Canola seed	6.00	6.00	6.00	7.00	7.00	7.00
Canola oil	3.05	3.05	1.90	2.20	1.65	0.80
Celite	2.00	2.00	2.00	-	-	-
Meat and bone meal 47 %	0.95	0.95	1.00	0.50	0.50	0.50
Limestone	0.79	0.79	0.79	0.79	0.79	0.80
Lysine HCL	0.435	0.435	0.440	0.420	0.420	0.390
Na Bicarbonate	0.325	0.325	0.330	0.315	0.315	0.305
DL-Methionine	0.295	0.295	0.250	0.250	0.245	0.175
Vitamin/Mineral Premix^b^	0.200	0.200	0.200	0.200	0.200	0.200
L-Threonine	0.155	0.155	0.145	0.135	0.135	0.095
L-Arginine	0.150	0.150	0.154	0.150	0.150	0.114
Sodium Chloride	0.130	0.130	0.130	0.115	0.115	0.125
L-Valine	0.086	0.086	0.062	0.064	0.062	0.028
L-Isoleucine	0.079	0.079	0.036	0.066	0.065	0.017
Choline Chloride	0.060	0.060	0.060	0.060	0.060	0.060
Xylanase	0.020	0.020	0.020	0.020	0.020	0.020
Phytase (2000 FYT/kg)	0.020	0.020	0.020	0.020	0.020	0.020
Cost AUD/ton	$647.3	$647.3	$622.4	$619.3	$610.7	$580.1
Calculated composition						
ME kcal/kg	3100	3100	3050	3150	3125	3100
Crude protein %	19.07	19.07	18.76	18.52	18.44	17.85
Dig.Lys %	1.08	1.08	1.06	1.03	1.02	0.96
Dig.Met %	0.55	0.55	0.50	0.50	0.50	0.42
Dig.M + C %	0.86	0.86	0.82	0.82	0.82	0.74
Dig.Thr %	0.72	0.72	0.70	0.69	0.68	0.62
Dig.Ile %	0.75	0.75	0.69	0.71	0.70	0.63
Dig.Leu %	1.18	1.18	1.16	1.15	1.14	1.11
Dig.Trp %	0.20	0.20	0.20	0.20	0.20	0.19
Dig.Arg %	1.18	1.18	1.16	1.13	1.12	1.05
Dig.His %	0.41	0.41	0.40	0.40	0.40	0.38
Dig.Val %	0.84	0.84	0.81	0.80	0.80	0.74
Crude Fat %	7.06	7.06	5.94	6.66	6.15	5.33
Crude Fibre %	3.60	3.60	3.61	3.84	3.85	3.85
Starch %	41.5	41.5	43.1	44.2	44.8	46.6
Ca %	0.75	0.75	0.75	0.70	0.70	0.70
Available P %	0.38	0.38	0.38	0.36	0.36	0.35
Total P %	0.42	0.42	0.42	0.40	0.40	0.40
Na %	0.19	0.19	0.19	0.18	0.18	0.18
Cl %	0.23	0.23	0.23	0.22	0.22	0.22
K %	0.67	0.67	0.65	0.65	0.65	0.63
DEB^c^ mEq/kg	188	188	185	183	182	177

a AGF: AgriFutures specifications.

b The premix contained (per kg of diet): [IU] retinol, 12,000; cholecalciferol, 5,000; [mg] tocopherol, 50; menadione, 3; thiamine, 3; riboflavin, 9; pyridoxine, 5; cobalamin, 0.025; niacin, 50; pantothenate, 10; folate, 2; biotin, 0.2; copper, 20; iron, 40; manganese, 110; cobalt, 0.25; iodine, 1; molybdenum, 2; zinc, 90; selenium, 0.3.

c Dietary electrolyte balance.

Diets were steam-pelleted at a conditioning temperature of 80 °C for 14 s. The pellet machine was equipped with a die ring with 4.0 mm holes and 38 mm thickness. The starter diets were further crumbled to maximize feed intake. The pellet durability index (PDI) of all diets was tested, in triplicate, using the NHP 200 New Holman Automatic Pellet Tester (TekPro Ltd, Norfolk, UK).

### Data collection

Birds were weighed on a pen basis on days 0, 10, 24, 35 and 42 to determine body weights (BW) and calculate body weight gain (BWG). Feed intake (FI) was measured in similar intervals and used to calculate FCR for each phase. Mortality was recorded daily, and the BW of dead birds was added to the total pen BW. Then pen BWG over each phase was calculated and used to determine the mortality corrected FCR for each pen. The overall FCR (0-42 d) was further corrected to a BW of 2.5 kg (FCRc) as there were treatment-associated differences in BW. This correction was achieved by considering a 50 g difference in BW was equivalent to 1 point (0.01) in FCR.

On day 35, 3 male and 3 female birds were euthanized by intravenous injection of sodium pentobarbitone and digesta from the distal ileum (defined as the second half of the ileum extending toward the cecal junction) was gently expressed and collected into sterile plastic containers. Digesta samples from birds within a pen were pooled, homogenized, freeze-dried and ground through a 0.5 mm screen. Digesta samples were analyzed for the content of the indigestible marker (Celite), protein, gross energy, starch and fat.

On day 41, all birds in each pen underwent visual inspection to assess footpad dermatitis and hock burns (lesions) on both feet. Any signs of footpad dermatitis were scored from 0 to 4 (Stracke et al., 2021), and hock burns were scored from 0 to 4 following the guidelines by (Kuttappan et al., 2016), as described in Xie et al. (2024).

On day 42, a total of 6 birds per pen (3 males and 3 females) with body weights close to the pen mean BW were randomly selected and euthanized by intravenous injection of sodium pentobarbitone for carcass analysis. Skinless breast meat (*pectoral* major and minor), leg quarter (thigh + drumstick), and abdominal fat pad were removed, weighed, and calculated as a percentage of live BW. Breast major muscles were also visually examined and scored for the occurrence of woody breast and white striping according to Xie et al. (2024).

For birds euthanized on days 35 and 42 for sample collection and carcass analysis, sex was determined post-mortem by visual examination of the gonads (presence of testes = male; absence of testes = female).

Diet prices, FI and FCR data were used to calculate feed cost per kg of live BW for each phase and the entire production period of 42 days. The average growth rate (g/b/day) was calculated for each treatment and used to calculate average age to 2.5 kg of live BW.

### Chemical analysis and calculations

Celite was included in the finisher diets at 20 g/kg as an inert marker. Celite concentrations were determined in triplicate and duplicate for diets and digesta samples respectively by the method of Siriwan et al. (1993). Starch concentration of diets and digesta were determined by the procedure based on the Megazyme Total Starch Assay method as described in Wang et al. (2025). The gross energy of diets and digesta were determined by bomb calorimetry using an adiabatic calorimeter (Parr 1281 bomb calorimeter, Parr Instruments Co., Moline, IL). Dry matter and fat contents of the diets and digesta were determined according to the methods of AOAC International (2005); dry matter was analyzed by oven drying at 105 °C (method 934.01), and fat content was determined using the ether extraction method (method 920.39).

The apparent digestibility coefficients for starch, fat, gross energy and protein in the ileal were calculated from the following equation:

$$Digestibility\;Coefficient=\;\frac{{\left(Nutrient/AIA\right)}_{diet}-{\left(Nutrient/AIA\right)}_{digesta}}{{\left(Nutrient/AIA\right)}_{diet}}$$

### Nutrient intake and conversion calculations

The ME (kcal/bird/day) and digestible lysine (g/bird/day) intake for each growth phase was calculated by multiplying the actual FI (g/b/day) by the corresponding calculated nutrient concentration based on the following equation:

NutrientIntake=FI/b/day×calculatednutrientconcerntration

A weighted average of ME and digestible lysine intake was also calculated for the entire production period of 0 to 42 days.

Conversion of ME and digestible lysine into live BW within each growth phase was calculated using the equation below:

$$Nutrient\;conversion=\frac{Total\;nutrient\;intake}{Body\;weight\;gain}$$

### Statistical analysis

Data were checked for normality and then subjected to statistical analysis using 2-way ANOVA of GLM procedure in JMP®13 (SAS Institute Inc., JMP Software, Cary, NC) to assess the main effects of diet nutrient density and genotype, and their interaction. Footpad dermatitis, hock burn, woody breast and white striping scores, recorded on an ordinal severity scale (0–4 or 0–3), were treated as quasi-continuous variables; mean pen scores for these traits were analyzed using the same linear model and are presented as mean scores per treatment. Carcass and nutrient digestibility data were analyzed as 3 × 2 × 2 factorial arrangement to assess the main effects of diet nutrient density, genotype and sex and their interaction. Each pen was considered as an experimental unit and the values presented in the tables are means with pooled standard error of the means (SEM). If a significant effect of treatment was detected, differences between treatments or main effects were separated by least square differences test (LSD) at *P* < 0.05.

## Results

Based on data presented in Table 3, on placement day, Cobb chicks were heavier than Ross by 1 g/b (*P* < 0.01). The genotype and nutrient specification of diets interacted for BW and BWG during the starter period (*P* < 0.05). The interaction resulted in lower BWG in Cobb birds fed diets formulated to AGF specs, while the diet specifications did not affect BWG of the Ross birds. As a main effect, Cobb birds gained around 15 g/b higher BW and consumed 38 g/b more feed than Ross, and as a result exhibited a higher FCR (*P* < 0.01). Feed intake and FCR were also affected by diet specifications, where birds fed diets based on Ross specs had lower FI (*P* < 0.05) and FCR (*P* < 0.01). Feed cost $/kg BW was significantly higher in Cobb birds but also lower with Cobb specs (*P* < 0.01).

**Table 3:** Broilers growth performance and feed cost per kg BW over the starter period (0–10 d).

Treatments^d^		BW g/b		BWG g/b	FI g/b	FCR g/g	Feed cost
Genotype	Diet	Day 0	Day 10	0–10 d	0–10 d	0–10 d	$/kg BW
Cobb	AGF	38.0	343^b^	305^bc^	354	1.161	0.76
Cobb	CB	38.1	358^a^	313^ab^	356	1.136	0.73
Cobb	RS	38.2	352^a^	314^a^	347	1.105	0.75
Ross	AGF	37.1	336^bc^	298^cd^	319	1.069	0.70
Ross	CB	37.2	331^c^	294^d^	316	1.076	0.69
Ross	RS	37.1	330^c^	293^d^	307	1.048	0.71
	SEM	0.21	3.01	3.02	3.60	0.009	0.006
*Main effects*							
Genotype							
Cobb		38.1^a^	349	311	352^a^	1.134^a^	0.75^a^
Ross		37.1^b^	332	295	314^b^	1.064^b^	0.70^b^
Diet							
AGF		37.6	339	302	336^a^	1.115^a^	0.73^b^
CB		37.7	341	304	336^a^	1.106^a^	0.71^b^
RS		37.6	341	305	327^b^	1.076^b^	0.73^b^
Source of variation *(P-value)*							
Genotype		<.001	<.001	<.001	<.001	<.001	<.001
Diet		0.817	0.752	0.776	0.015	0.001	0.007
Genotype × Diet		0.652	0.037	0.040	0.767	0.139	0.151

Each value for each treatment represents the mean of 8 replicates of 45 birds each.

a-c Means within a column not sharing a superscript differ significantly at the *P* < 0.05 level for the treatment effects and at the P level shown for the main effects.

d AGF: AgriFuture specs, CB: Cobb specs, RS: Ross specs.

Over the grower period, an interaction between genotype and diet specs resulted in higher day 24 BW of Cobb birds fed the Ross specs, while Ross birds fed the Cobb specs recorded the lowest BW (Table 4; *P* < 0.01). Bodyweight gain during this period was affected by both genotype and diets specs, where Cobb birds gained higher weight than Ross, and Ross specs resulted in higher BWG compared to AGF and Cobb (*P* < 0.01). Cobb birds fed Cobb specs recorded the highest FI, while Ross birds fed the Cobb specs recorded the lower FI, resulting in an interaction between diet specs and genotype (*P* < 0.05). Genotype and diet specs independently affected FCR, being higher in Cobb than Ross, and the lowest FCR was observed in birds fed Ross specs compared to both AGF and Cobb (*P* < 0.01). Following the trend in the starter period, feed cost $/kg BW was significantly higher in Cobb birds, also Cobb specs resulted in higher feed cost $/kg BW (*P* < 0.01).

**Table 4:** Broilers growth performance and feed cost per kg BW over the grower period (10–24 d).

Treatments^d^		BW g/b	BWG g/b	FI g/b	FCR g/g	Feed cost
Genotype	Diet	Day 24	10-24 d	10-24 d	10-24 d	$/kg BW
Cobb	AGF	1465^b^	1123	1551^b^	1.381	0.87
Cobb	CB	1482^ab^	1130	1624^a^	1.437	0.88
Cobb	RS	1511^a^	1159	1550^b^	1.338	0.87
Ross	AGF	1384^c^	1049	1415^c^	1.349	0.85
Ross	CB	1355^d^	1022	1432^c^	1.401	0.86
Ross	RS	1383^cd^	1052	1375^d^	1.306	0.85
	SEM	10.6	9.3	11.2	0.005	0.004
*Main effects*						
Genotype						
Cobb		1486	1137^a^	1575	1.386^a^	0.87^a^
Ross		1373	1041^b^	1407	1.352^b^	0.85^b^
Diet						
AGF		1425	1085^b^	1483	1.365^b^	0.86^b^
Cobb		1418	1076^b^	1528	1.419^a^	0.87^a^
Ross		1447	1105^a^	1462	1.322^c^	0.86^b^
Source of variation *(P-value)*						
Genotype		<.001	<.001	<.001	<.001	<0.001
Diet		0.025	0.010	<.001	<.001	0.001
Genotype × Diet		0.047	0.129	0.042	0.911	0.956

Each value for each treatment represents the mean of 8 replicates of 45 birds each.

a-c Means within a column not sharing a superscript differ significantly at the *P* < 0.05 level for the treatment effects and at the P level shown for the main effects.

d AGF: AgriFuture specs, CB: Cobb specs, RS: Ross specs.

As summarized in Table 5, there was no interaction between genotype and diet specs for any performance parameters during the finisher phase (24–35 d; *P* > 0.05). However, as the main effect, Cobb birds recorded higher BW on day 35, regardless of the sex (*P* < 0.01). In addition, independent of genotype, birds fed Ross specs gained lower weight during the finisher period (*P* < 0.01). Feed intake followed similar patterns, being higher in Cobb birds and lower in birds fed Ross specs (*P* < 0.01). Cobb birds recorded the highest FCR (*P* < 0.01), and birds fed AGF specs recorded statistically lower FCR than both Ross and Cobb specs (*P* < 0.05). Over the finisher period, the feed cost $/kg BW remained higher in Cobb birds (*P* < 0.01). Cobb specs resulted in the lower feed cost while Ross specs resulted in the highest feed cost $/kg BW (*P* < 0.01).

**Table 5:** Broilers growth performance and feed cost per kg BW over the finisher period (24–35 d).

Treatments^d^		BW g/b	BW g/b	BW g/b	BWG g/b	FI g/b	FCR g/g	Feed cost
Genotype	Diet	Day 35	Female e	Male e	24–35 d	24–35 d	24–35 d	$/kg BW
Cobb	AGF	2742	2457	2969	1276	1925	1.509	0.98
Cobb	CB	2774	2473	2918	1292	1983	1.536	0.96
Cobb	RS	2728	2512	2963	1217	1886	1.550	1.00
Ross	AGF	2553	2284	2829	1168	1720	1.472	0.95
Ross	CB	2529	2261	2727	1176	1747	1.486	0.92
Ross	RS	2543	2338	2790	1161	1708	1.471	0.95
	SEM	19.0	47.5	40.8	13.3	14.2	0.008	0.006
*Main effects*								
Genotype								
Cobb		2748^a^	2481^a^	2950^a^	1262^a^	1931.7^a^	1.532^a^	0.98^a^
Ross		2542^b^	2294^b^	2782^b^	1168^b^	1725.4^b^	1.476^b^	0.94^b^
Diet								
AGF		2647	2370	2899	1222^a^	1823^b^	1.491^b^	0.96^b^
Cobb		2652	2367	2823	1234^a^	1865^a^	1.511^a^	0.94^c^
Ross		2636	2425	2876	1189^b^	1797^b^	1.510^a^	0.98^a^
Source of variation *(P-value)*								
Genotype		<.001	<.001	<.001	<.001	<.001	<.001	<0.001
Diet		0.687	0.394	0.170	0.005	<.001	0.041	<0.001
Genotype × Diet		0.227	0.893	0.824	0.064	0.139	0.064	0.053

Each value for each treatment represents the mean of 8 replicates of 45 birds each.

a-c Means within a column not sharing a superscript differ significantly at the *P* < 0.05 level for the treatment effects and at the P level shown for the main effects.

d AGF: AgriFuture specs, CB: Cobb specs, RS: Ross specs.

e Sex-specific body weights were derived from 6 sampled birds per replicate; consequently, these values should not be interpreted as the actual counts or proportions of females and males within each replicate.

On day 42, Cobb birds, regardless of their sex, recorded higher BW than Ross bird (Table 6; *P* < 0.01). However, BWG during the withdrawal phase was neither affected by genotype nor diet specs, though numerically (20 g/b) was higher in Ross birds (*P* > 0.05). Cobb birds consumed more feed than Ross, and as a result had a higher FCR during the withdrawal period (*P* < 0.01). In addition, birds fed Cobb specs had the highest FCR (*P* < 0.05). During this phase, following the same trend from other phases, feed cost $/kg BW was higher in Cobb birds than Ross (*P* < 0.01). Cobb specs also resulted in lower feed cost $/kg BW when compared to AGF and Ross specs (*P* < 0.01).

**Table 6:** Broilers growth performance and feed cost per kg BW over the withdrawal period (35–42 d).

Treatments^d^		BW g/b			BWG g/b			FI g/b	FCR g/g
Genotype	Diet	Day 42	Female^e^	Male^e^	35–42 d	Female^e^	Male^e^	35–42 d	35–42 d
Cobb	AGF	3519	3210	3938	777	753	968	1430	1.843
Cobb	CB	3547	3160	3957	773	686	1038	1478	1.913
Cobb	RS	3508	3199	3932	780	687	969	1449	1.859
Ross	AGF	3345	2969	3719	792	685	889	1373	1.736
Ross	CB	3325	2975	3804	795	714	1075	1405	1.770
Ross	RS	3339	2963	3709	795	625	919	1374	1.730
	SEM	17.9	60.5	61.6	12.9	69.2	69.4	19.6	0.018
*Main effects*									
Genotype									
Cobb		3525^a^	3190^a^	3942^a^	776	708.9	992	1452^a^	1.872^a^
Ross		3336^b^	2969^b^	3744^b^	794	674.9	962	1384^b^	1.745^b^
Diet									
AGF		3432	3090	3828	784	719.3	929	1402	1.790^b^
Cobb		3436	3068	3880	784	700.4	1057	1442	1.841^a^
Ross		3423	3082	3820	787	656.0	944	1411	1.795^b^
Source of variation *(P-value)*									
Genotype		<.001	<.001	0.001	0.108	0.551	0.594	<.001	<.001
Diet		0.776	0.934	0.579	0.957	0.647	0.142	0.116	0.015
Genotype × Diet		0.265	0.878	0.818	0.956	0.743	0.688	0.894	0.622

Each value for each treatment represents the mean of 8 replicates of 45 birds each.

a-c Means within a column not sharing a superscript differ significantly at the *P* < 0.05 level for the treatment effects and at the P level shown for the main effects.

d AGF: AgriFuture specs, CB: Cobb specs, RS: Ross specs.

e Sex-specific body weights and bodyweight gains were derived from 6 sampled birds per replicate; consequently, these values should not be interpreted as the actual counts or proportions of females and males within each replicate.

Table 7 summarizes broilers key performance metrices determined for the overall grow-out period of 0–42 days. There was no interactive effect of genotype or diet specs on BWG over the entire production period (*P* > 0.05). Irrespective of diet specs, Cobb birds on average gained 182 g/bird higher BW than Ross (*P* < 0.01). Feed intake during the same period was affected by both genotype and diet specs (*P* < 0.01), where Cobb birds had a higher intake than Ross, and birds fed diets based on Cobb specs consumed more feed than AGF or Ross. FCR also followed the same trends, and was higher in Cobb than Ross, and higher with Cobb specs than AGF or Ross (*P* < 0.01). The same pattern for FCR was maintained when the FCR values were corrected to 2.5 kg of BW, however, the magnitude of differences between Cobb and Ross reduced from 6 points to 2 points when FCR was corrected for BW. The age to reach 2.5 kg of BW was only affected by genotype and Cobb birds grew faster by an average of 1.7 days to get to 2.5 kg of BW (*P* < 0.01). Food pad dermatitis was affected neither by genotype nor the diet specs, or their interactions (*P* > 0.05). However, Cobb birds had a significantly higher hock burns score (*P* < 0.01). Over the entire production period of 42 days, an interaction between genotype and diet specs resulted in higher feed cost $/kg BW in Cobb birds fed Ross specs compared to Ross birds fed Ross specs, but with Cobb specs Ross birds recorded the lowest feed cost $/kg BW and feed cost $/bird (*P* < 0.01). The overall mortality in the current study was not affected by dietary treatments.

**Table 7:** Broilers growth performance over the entire feeding trial (0–42 d), age to 2.5 kg and feed cost per kg BW and per bird, foot pad dermatitis (FD) and hock burns (HB).

Treatments^d^		BWG g/b	FI g/b	FCR g/g	cFCR^e^ g/g	Age to	Foot pad^f^		Feed cost		
Genotype	Diet	0–42 d	0–42 d	0–42 d	0–42 d	2.5 kg	FD	HB	$/kg	$/b	
Cobb	AGF	3482	5261	1.511	1.315	30.2	1.51	2.16	0.96	3.34	
Cobb	CB	3501	5435	1.553	1.352	30.0	1.30	1.68	0.94	3.32	
Cobb	RS	3470	5232	1.508	1.314	30.3	1.02	1.73	0.95	3.34	
Ross	AGF	3315	4834	1.458	1.296	31.7	1.02	1.10	0.93	3.06	
Ross	CB	3288	4902	1.491	1.333	31.9	1.18	1.14	0.91	2.98	
Ross	RS	3303	4765	1.443	1.282	31.8	0.93	1.32	0.92	3.04	
	SEM	16.8	23.6	0.0057	0.0078	0.15	0.215	0.197	0.004	0.015	
*Main Effects*											
Genotype											
Cobb		3484^a^	5310^a^	1.524^a^	1.327^a^	30.1^a^	1.28	1.86^a^	0.95^a^		3.33^a^
Ross		3302^b^	4834^b^	1.464^b^	1.304^b^	31.8^b^	1.04	1.18^b^	0.92^b^		3.03^b^
Diet											
AGF		3398	5048^b^	1.485^b^	1.305^b^	30.9	1.26	1.63	0.94^a^		3.20^a^
Cobb		3395	5169^a^	1.522^a^	1.343^a^	31.0	1.24	1.41	0.93^b^		3.15^b^
Ross		3386	4999^c^	1.475^b^	1.298^b^	31.0	0.97	1.52	0.94^a^		3.19^a^
Source of variation *(P-value)*											
Genotype		<.001	<.001	<.001	0.001	<.001	0.153	0.001	<.001		<.001
Diet		0.772	<.001	<.001	<.001	0.760	0.241	0.485	<.001		0.004
Genotype × Diet		0.302	0.090	0.540	0.666	0.287	0.550	0.188	0.690		0.188

Each value for each treatment represents the mean of 8 replicates of 45 birds each. The number of birds examined for FD and HB may be slightly lower in some pens due to mortality.

a-c Means within a column not sharing a superscript differ significantly at the *P* < 0.05 level for the treatment effects and at the P level shown for the main effects.

d AGF: AgriFuture specs, CB: Cobb specs, RS: Ross specs.

e FCR corrected to a body weight of 2.5 kg.

f Scores for footpad dermatitis and hock burns used a 0 to 4 scale (0: no lesion; 4: severe lesions).

There were no 3-way interactions of the experimental factors for any of the carcass yield components or breast fillet quality determined on day 42 (Table 8; *P* > 0.05). Breast meat yield was not affected by genotype or diet specs (*P* > 0.05). However, sex as a main factor affected yield component of the breast, where female birds recorded lower major, minor and total breast fillet yield than males (*P* < 0.05). Ross birds recorded a higher leg quarter yield than Cobb (*P* < 0.05). Fat pad weight as a percentage of live BW was higher in Cobb than Ross, and also higher in females than males (*P* < 0.01). While woody breast scores were not affected by genotype or diet specs, females had a lower woody breast score than males (*P* < 0.01). White stripping scores were significantly higher in Cobb birds than Ross, and in males than females (*P* < 0.01).

**Table 8:** Carcass yield components, woody breast (WB) and white stripping (WS) scores recorded on day 42.

Treatments^d^	Carcass Yield g/100 g live BW^e^			Breast Score^f^					
Genotype	Diet	Sex	*P*. major	*P*. minor	*P*. total	Leg Qtr.	Fat pad	WB	WS
Cobb	AGF	F	21.1	4.28	25.4	19.0	1.60	1.95	0.86
Cobb	AGF	M	22.0	3.94	25.9	19.0	1.29	2.70	1.34
Cobb	CB	F	20.7	4.20	24.9	19.0	1.59	2.08	0.82
Cobb	CB	M	21.6	3.87	25.5	19.3	1.35	2.66	1.34
Cobb	RS	F	20.8	4.18	25.0	18.9	1.69	1.85	0.90
Cobb	RS	M	21.7	3.82	25.5	19.3	1.37	2.64	1.25
Ross	AGF	F	21.1	4.21	25.3	19.1	1.40	1.78	0.38
Ross	AGF	M	22.0	3.87	25.9	19.6	1.12	2.44	0.80
Ross	CB	F	20.8	4.15	24.9	19.4	1.45	1.74	0.38
Ross	CB	M	21.6	3.79	25.4	19.6	1.11	2.57	0.76
Ross	RS	F	20.8	4.10	24.9	19.4	1.47	1.72	0.30
Ross	RS	M	21.7	3.77	25.5	19.4	1.21	2.34	0.84
	SEM		0.27	0.063	0.29	0.18	0.049	0.171	0.148
*Main effects*									
Genotype									
Cobb			21.3	4.05	25.3	19.1^b^	1.48^a^	2.31	1.08^a^
Ross			21.3	3.98	25.3	19.4^a^	1.30^b^	2.10	0.58^b^
									
Diet									
AGF			21.5	4.08	25.6	19.2	1.35	2.22	0.84
Cobb			21.2	4.00	25.2	19.3	1.37	2.26	0.82
Ross			21.3	3.97	25.2	19.2	1.44	2.14	0.82
Sex									
Female			20.9^b^	4.19^a^	25.1^b^	19.1	1.53^a^	1.85^b^	0.60^b^
Male			21.8^a^	3.84^b^	25.6^a^	19.4	1.24^b^	2.56^a^	1.06^a^
Source of variation *(P-value)*									
Genotype			0.855	0.167	0.897	0.017	<.001	0.100	<.001
Diet			0.316	0.166	0.207	0.718	0.184	0.726	0.985
Sex			<.001	<.001	0.018	0.117	<.001	<.001	0.001
Genotype × Diet × Sex			0.998	0.973	0.995	0.452	0.663	0.786	0.818

Each value for each treatment represents the mean of 8 replicates with 6 birds per replicate (3 males + 3 females) sacrificed for carcass yield data.

a-c Means within a column not sharing a superscript differ significantly at the *P* < 0.05 level for the treatment effects and at the P level shown for the main effects.

d AGF: AgriFuture specs, CB: Cobb specs, RS: Ross specs.

e P. major: Pectoralis major (breast fillet); P. minor: Pectoralis minor (tenderloin); P. total: Pectoral total (combined breast fillet + tenderloin); Leg Qtr: leg quarter (thigh + drumstick).

f Woody breast and white striping were scored on a 0 to 3 scale (WB: 0 = no WB, 1 = moderate, 2 = severe, 3 = extreme; WS: 0 = normal, 1 = moderate, 2 = severe, 3 = extreme).

According to the data presented in Table 9, ileal digestibility coefficient of protein, gross energy, starch and fat was not affected by an interaction between genotype, diet or sex (*P* > 0.05). Female birds had a significantly lower protein digestibility coefficient than male birds, irrespective of diet or genotype (*P* < 0.01). There was no significant three-way interaction among genotype, diet, and sex on ileal digestibility of GE. However, diets formulated to Cobb specifications resulted in lower GE digestibility (*P* < 0.01). Sex also influenced energy utilization, with female birds showing significantly lower GE digestibility than males (*P* < 0.01). Ileal starch digestibility was not affected by any of the experimental factors or their interaction (*P* > 0.05). Fat digestibility was only affected by diet and sex, where Cobb specifications resulted in lower fat digestibility and female birds recorded lower fat digestibility than males (*P* < 0.01).

**Table 9:** Ileal digestibility coefficient of protein, gross energy (GE), starch and fat determined on day 35 of the experiment.

Treatments^d^			Digestibility coefficient			
Genotype	Diet	Sex	Protein	GE	Starch	Fat
Cobb	AGF	F	0.725	0.706	0.984	0.943
Cobb	AGF	M	0.812	0.804	0.979	0.955
Cobb	CB	F	0.677	0.640	0.981	0.918
Cobb	CB	M	0.804	0.764	0.975	0.941
Cobb	RS	F	0.702	0.691	0.975	0.938
Cobb	RS	M	0.800	0.792	0.976	0.949
Ross	AGF	F	0.726	0.707	0.983	0.940
Ross	AGF	M	0.848	0.825	0.982	0.959
Ross	CB	F	0.719	0.667	0.979	0.925
Ross	CB	M	0.799	0.758	0.979	0.933
Ross	RS	F	0.715	0.694	0.980	0.934
Ross	RS	M	0.825	0.809	0.973	0.953
	SEM		0.015	0.014	0.003	0.005
						
*Main effects*						
Genotype						
Cobb			0.753	0.732	0.978	0.941
Ross			0.771	0.743	0.979	0.941
						
Diet						
AGF			0.777	0.760^a^	0.981	0.949^a^
Cobb			0.749	0.707^b^	0.978	0.929^b^
Ross			0.760	0.746^a^	0.976	0.944^a^
						
Sex						
Female			0.710^b^	0.684^b^	0.980	0.933^b^
Male			0.814^a^	0.791^a^	0.977	0.948^a^
						
Source of variation *(P-value)*						
Genotype			0.128	0.332	0.652	0.991
Diet			0.166	0.004	0.152	0.001
Sex			<.001	<.001	0.192	0.001
Genotype × Diet × Sex			0.368	0.547	0.508	0.404

Each value for each treatment represents the mean of 8 replicates with 6 birds per replicate (3 males + 3 females) sacrificed for digestibility analysis.

a-c Means within a column not sharing a superscript differ significantly at the *P* < 0.05 level for the treatment effects and at the P level shown for the main effects.

d AGF: AgriFuture specs, CB: Cobb specs, RS: Ross specs.

Nutrient intake calculations presented in Table 10 revealed a significant interaction between genotype and diet for ME intake only during the grower phase (*P* < 0.05), indicating that Ross birds had similar ME intakes regardless of diet, whereas Cobb birds fed the AGF diet consumed significantly less ME compared to Cobb birds fed Cobb or Ross diets. In all other growth phases, genotype had a significant effect on nutrient intake, with Cobb birds consistently showing higher intakes of both ME and digestible lysine (*P* < 0.05). Diet also influenced digestible lysine intake, with birds fed the AGF diet tending to consume the same or more than those on the Cobb or Ross diets (*P* < 0.05).

**Table 10:** Metabolizable energy (ME) and digestible lysine intake within each phase.

Treatment^d^		ME intake (kcal/bird/day)					Digestible Lys Intake (g/bird/day)				
Genotype	Diet	STR	GRW	FIN	WDRL	0–42 d	STR	GRW	FIN	WDRL	0–42 d
Cobb	AGF	101.8	330^b^	543	644	384	0.468	1.31	1.89	2.11	1.393
Cobb	CB	103.3	342^a^	550	655	392	0.449	1.35	1.91	2.03	1.394
Cobb	RS	103.2	338^a^	532	647	384	0.458	1.31	1.85	2.11	1.381
Ross	AGF	91.7	301^c^	485	618	341	0.421	1.19	1.69	2.02	1.277
Ross	CB	91.8	302^c^	484	623	353	0.399	1.19	1.68	1.93	1.253
Ross	RS	91.4	299^c^	481	614	350	0.405	1.16	1.68	2.00	1.256
	SEM	1.04	2.4	4.0	8.8	4.7	0.0047	0.009	0.014	0.028	0.006
*Main effects*											
Genotype											
Cobb		102.8^a^	336	541^a^	649^a^	387^a^	0.458^a^	1.32^a^	1.88^a^	2.08^a^	1.389^a^
Ross		91.6^b^	301	484^b^	618^b^	348^b^	0.408^b^	1.18^b^	1.68^b^	1.98^b^	1.262^b^
Diet											
AGF		96.8	315	514^ab^	631	362	0.444^a^	1.25^ab^	1.79	2.06^a^	1.335^a^
Cobb		97.5	322	517^a^	639	372	0.424^b^	1.27^a^	1.80	1.98^b^	1.323^ab^
Ross		97.3	319	506^b^	630	367	0.432^b^	1.23^b^	1.76	2.06^a^	1.319^a^
											
Source of variation *(P-value)*											
Genotype		<0.001	<0.001	<0.001	<0.001	<0.001	<0.001	<0.001	<0.001	<0.001	<0.001
Diet		0.759	0.023	0.031	0.569	0.110	<0.001	0.004	0.056	0.006	0.035
Genotype × Diet		0.680	0.044	0.174	0.902	0.685	0.800	0.053	0.180	0.912	0.135

Each value for each treatment represents the mean of 8 replicates of 45 birds each.

a-c Means within a column not sharing a superscript differ significantly at the *P* < 0.05 level for the treatment effects and at the P level shown for the main effects.

d AGF: AgriFuture specs, CB: Cobb specs, RS: Ross specs.

There were no interactive effects of genotype and diet on nutrient utilization during any of the growth phases (Table 11; *P* > 0.05). Cobb birds consistently recorded a higher ME and digestible lysine conversion value than Ross (*P* < 0.01). Diet specs influenced ME utilization differently within the grower and finisher phases, although it had no significant effect on overall ME conversion ratio (*P* > 0.05). Digestible lysine conversion ratio was affected by diet specs within all four growing phases (*P* < 0.01). From day 0 to 42, AGF specs resulted in higher digestible lysine conversion ratio compared to the Cobb and Ross specs (*P* < 0.05).

**Table 11:** Metabolizable energy and digestible lysine conversion to kg body weight within each phase.

Treatment^d^		ME conversion (Mcal/kg BW)					Digestible Lys conversion (g/kg BW)				
Genotype	Diet	STR	GRW	FIN	WDRL	0–42 d	STR	GRW	FIN	WDRL	0–42 d
Cobb	AGF	3.34	4.11	5.95	11.61	4.63	15.3	16.3	20.7	38.0	16.8
Cobb	CB	3.29	4.24	5.96	11.86	4.69	14.3	16.7	20.7	36.7	16.7
Cobb	RS	3.29	4.08	6.11	11.62	4.65	14.6	15.8	21.3	37.9	16.7
Ross	AGF	3.07	4.01	5.81	10.94	4.33	14.1	15.9	20.2	35.8	16.2
Ross	CB	3.12	4.13	5.77	10.97	4.51	13.6	16.3	20.1	34.0	16.0
Ross	RS	3.12	3.98	5.80	10.81	4.45	13.8	15.4	20.2	35.3	16.0
	SEM	0.028	0.017	0.034	0.116	0.060	0.127	0.068	0.120	0.374	0.062
*Main effects*											
Genotype											
Cobb	3.31^a^	4.14^a^	6.01^a^	11.70^a^	4.66^a^		14.7^a^	16.3^a^	20.9^a^	37.5^a^	16.7^a^
Ross	3.10^b^	4.04^b^	5.79^b^	10.91^b^	4.43^b^		13.8^b^	15.9^b^	20.2^b^	35.0^b^	16.1^b^
Diet											
AGF	3.21	4.06^b^	5.88^b^	11.28	4.48		14.7^a^	16.1^b^	20.5^b^	36.9^a^	16.5^a^
Cobb	3.21	4.19^a^	5.87^b^	11.42	4.60		13.9^c^	16.5^a^	20.4^b^	35.4^b^	16.4^b^
Ross	3.20	4.03^b^	5.96^a^	11.22	4.55		14.2^b^	15.6^c^	20.8^a^	36.6^a^	16.4^b^
Source of variation *(P-value)*											
Genotype	<0.001	<0.001	<0.001	<0.001	<0.001		<0.001	<0.001	<0.001	<0.001	<0.001
Diet	0.984	<0.001	0.022	0.227	0.141		<0.001	<0.001	0.010	<0.001	0.015
Genotype × Diet	0.165	0.937	0.059	0.654	0.595		0.114	0.932	0.059	0.748	0.448

Each value for each treatment represents the mean of 8 replicates of 45 birds each.

a-c Means within a column not sharing a superscript differ significantly at the *P* < 0.05 level for the treatment effects and at the P level shown for the main effects.

d AGF: AgriFuture specs, CB: Cobb specs, RS: Ross specs.

## Discussion

Regardless of dietary specifications, both Ross and Cobb birds in this study outperformed their respective breeder guidelines for straight-run broilers (Aviagen, 2022a; Cobb Vantress, 2022), achieving higher body weights and, despite greater feed intake than breeder objectives, demonstrated superior FCR. This study served both as a validation trial and an investigation into the performance responses of Cobb and Ross broiler chickens to dietary nutrient density. In our previous studies, we observed that younger Ross birds are less responsive to changes in dietary energy density (Toghyani et al., 2024; Xie et al., 2024; Wang et al., 2025) compared to amino acid concentrations, when diets are formulated based on the concept of balanced protein or ideal amino acid ratios (Toghyani et al., 2024). However, all the previous studies were conducted using male Ross 308 chicks, where a reduction of 100 kcal/kg in the starter diet and 75 kcal/kg in the grower diet, relative to breeder recommendations (Aviagen, 2022a), did not adversely affect growth rate or feed efficiency.

According to breeder specifications, both ME and amino acid densities are lower for Cobb (Cobb Vantress, 2022) than for Ross (Aviagen, 2022a). Additionally, Cobb and Ross birds follow different growth trajectories (Benyi et al., 2015; Nogueira et al., 2019; Tona et al., 2010). As also observed in this study, Cobb 500 broilers generally exhibit a faster early growth rate, especially in the starter phase, making them suitable for short-cycle production systems. In contrast, Ross 308 birds tend to grow more steadily across production phases and may slightly lag in early growth but typically catch up by market age. Ross 308, developed by Aviagen in the United Kingdom, has been selectively bred for rapid growth, high feed efficiency, and balanced carcass composition. Due to its development in a region where wheat is a predominant grain source, Ross 308 has been reported to perform better on wheat-based diets when compared to maize-based diets (Chrystal et al., 2020; Yang et al., 2020). In contrast, Cobb 500, developed by Cobb Vantress in the United States, has been selected primarily using maize-based diets. This difference in dietary adaptation may partially explain variations in nutrient specifications and growth trajectories between the two Genotypes.

Throughout the 42-day production period, dietary nutrient specifications did not significantly affect growth rate, resulting in comparable body weights at day 42 across all nutrient density treatments. These findings are consistent with our previous observations, where reducing dietary ME or increasing amino acid density did not influence growth rate or final body weight (Xie et al., 2024). However, the lower ME and AA density in the Cobb specifications resulted in higher feed intake compared to both AGF and Ross specifications, irrespective of the Genotype. Birds fed the AGF diets, which also had lower ME in starter and grower had a higher feed intake compared to the Ross diets, but this did not translate into compromised FCR (0-42 d). In the current study, irrespective of diet nutrient density, Cobb broilers grew faster and achieved greater body weights than Ross. Cobb chicks were heavier at the beginning of the study (day 0) and showed higher early-stage growth rates. However, the higher feed intake in Cobb broilers resulted in a poorer FCR compared to Ross. This trend may indicate a shift in feeding behavior, where modern broiler chickens may no longer regulate feed intake to meet their most limiting nutrient, but are instead limited by their physical capacity to consume feed (Aftab, 2019). It is worth noting that females appear to be more sensitive to changes in dietary nutrient density and may adjust their feed intake accordingly. In the study by Muñoz et al. (2023), the authors reported that females showed a more pronounced reduction in growth performance and feed intake in response to lower nutrient density compared to males. This suggests that females may have less capacity to compensate for nutrient dilution through increased feed intake, making them more nutritionally sensitive. This may partly explain the higher FCR in starter and grower phases in response to AGF (lower ME) specifications than Ross, compared to our previous studies (Toghyani et al., 2024; Xie et al., 2024; Wang et al., 2025) where male birds were utilized and as such the lower ME density had no effect on feed efficiency. Previous studies have also shown that Cobb broilers fed diets with varying ME levels maintained similar body weights, but higher FI was noted in low ME diets (Dozier and Gehring, 2014). Overall, Cobb birds recorded an FCR approximately 6 points higher than Ross. However, when FCR was corrected for a BW of 2.5 kg, the difference decreased to 2.5 points. Still, Cobb specifications resulted in the highest FCR, while AGF and Ross specifications yielded comparable FCR, both raw and corrected.

Although previous studies have reported inconsistent results when comparing Cobb and Ross broilers, but similar to the findings of the current study, Cobb broilers generally achieved higher final weights by day 42 (Benyi et al., 2015; Dozier and Gehring, 2014; Tona et al., 2010), with the exception of Nogueira et al. (2019) who reported higher final weights in Ross. These discrepancies may arise from differences in basal diet composition, particularly the primary grain used (wheat vs. maize). Differences between Cobb and Ross broilers are evident even during embryonic development. Tona et al. (2010) noted that Cobb eggs tended to hatch earlier, with higher resonate frequencies and a faster decline, indicating accelerated development. Heat production was also significantly higher in Cobb chicks post-hatch. However, when Druyan (2010) compared embryonic development of the two genotypes, Ross embryos showed higher oxygen consumption and heart rates suggesting increased metabolic levels compared to their Cobb counterparts. Despite these findings showing conflicting results, both studies show that genetic differences between the genotypes not only influence growth performance but also metabolic efficiency and developmental trajectories (Druyan, 2010; Tona et al., 2010).

Body weight gain during the withdrawal period (35–42 d) was not significantly different between the genotypes, although Ross broilers gained numerically more weight (∼20 g/bird). Therefore, despite Cobb’s rapid early growth, the two genotypes may ultimately reach comparable final weights if the trial period was extended, albeit at different rates. This shows the importance of considering genotype-specific growth curves when formulating diets for different broiler genotypes. Male chicks across both genotypes recorded higher body weight gains when fed the Cobb diet in the withdrawal period, suggesting that males may respond more favorably to lower ME diets by increasing their intake and consequently ingesting more amino acids. However, female broilers tend to reach their peak body weight earlier than male counterparts and begin depositing fat at an earlier age, as also observed in this study. This pattern is influenced by both physiological and hormonal factors associated with natural reproductive development in female birds (Benyi et al., 2015; Tumová and Teimouri, 2010).

The higher BW observed in Cobb birds is likely a contributing factor to the increased incidence of hock burns. While footpad dermatitis is primarily associated with decreasing litter quality, particularly increased moisture content, hock burns have been shown to correlate more directly with body weight, as heavier birds are prone to spending extended periods sitting (Van den Oever et al., 2020). Consistent with our results, Çapar Akyüz et al. (2022) reported higher hock dermatitis in fast-growing broilers irrespective of sex, with the highest frequencies observed in both fast-growing males and females. Similarly, Van der Eijk et al. (2023) reported that fast-growing broilers had poorer littler conditions and a greater prevalence of skin lesions.

Existing literature suggests that nutrient digestibility coefficients are influenced by both feed intake and dietary nutrient concentrations (Teeter and Smith, 1985; Toghyani et al., 2025). Higher feed intake has been reported to be associated with reduced nutrient digestibility, as it increases the rate of passage through the gut, leaving less time for enzymatic digestion and nutrient absorption; additionally, elevated intake can dilute the concentration of digestive enzymes per unit of digesta, further lowering digestion efficiency, particularly for fat and protein (Svihus, 2011). In the current study, although Cobb broilers consumed more feed than Ross, there were no significant differences in the digestibility coefficients of protein, energy, starch, or fat between the two genotypes. However, nutrient density had a significant effect on digestibility where birds fed the Ross and AGF diets exhibited higher energy and fat digestibility than those fed the Cobb specs. As starch digestibility was not influenced by diet specification, the reduced GE digestibility observed with the Cobb specifications could also likely be attributed to the lower fat digestibility in spite of the Cobb diet being lower in crude fat. The variation in fat digestibility may be linked to physiological adaptations in the digestive system. Pancreatic enzyme activity is known to respond to both the type and amount of dietary substrate. Studies in rats (Snook, 1971; Birk et al., 2014) and pigs (Corring, 1980) have demonstrated that pancreatic secretions are dynamically regulated by diet composition. Birk et al. (2014) found that high-fat diets upregulated mRNA expression of pancreatic enzymes associated with lipid digestion. A similar adaptive capacity likely exists in broilers. Birds fed low ME and low-fat diets from hatch (Cobb specs) may have reduced pancreatic stimulation, consistent with the principle of enzyme economy, where enzymatic output is modulated by nutrient availability. Additionally, the digestive tract can undergo morphological adaptations in response to dietary fat levels. For example, Petit et al. (2007) reported that high lipid diets upregulated genes related to lipid transport, absorption, metabolism, and increased proliferative activity in the jejunal mucosa in mice. These findings suggest that dietary fat levels not only regulate enzyme secretion but also alter the absorptive and structural capacity of the small intestine. Furthermore, within this context, the role of endogenous fat losses should also be considered. Regardless of dietary fat content, endogenous fats are continually secreted into the gastrointestinal tract, contributing to the fat present in ileal digest (Ravindran et al., 2016; Toghyani et al., 2025). In broiler chickens, endogenous fat losses have been estimated at approximately 1714 mg/kg of DM intake (Tancharoenrat et al., 2014). In low-fat diets, the absolute fat entering the gut is minimal, while endogenous losses remain relatively constant. As a result, these losses represent a greater proportion of total fat in the digesta, leading to an overestimation of undigested dietary fat when not corrected. Conversely, high-fat diets dilute the relative impact of endogenous fat losses, resulting in higher apparent fat digestibility (Birk et al., 2014; Jørgensen et al., 1993). In this study, fat digestibility was measured on day 35, when both Ross and AGF finisher diets had substantially higher supplemental fat inclusion. These factors may therefore help explain the lower fat digestibility observed with the Cobb specifications, which generally included less supplemental fat in the finisher phase. Across the entire production period (0-42 d), Cobb diets resulted in numerically higher ME intake and ME conversion per kilogram of body weight, consistent with the observed trends in GE and fat digestibility. Providing energy beyond what is required to support optimal protein accretion does not necessarily improve energy digestibility or growth performance, instead, it can promote excess fat deposition (Collin et al., 2003; Toghyani et al., 2025; Xie et al., 2024).

Furthermore, sex-based differences in digestibility were evident in this study, female birds consistently exhibited lower protein, energy and fat digestibility coefficients compared to males. These differences likely stem from inherent differences in gut microbiota, metabolic rate, growth patterns, and endocrine functions between the sexes (Cui et al., 2021; Decuypere et al., 2007; England et al., 2023). Despite ongoing efforts to understand the role of gut microbiota, current literature on microbiota abundance within the gut and ceca between the sexes remains inconsistent (Cui et al., 2021; England et al., 2024; Wang et al., 2021). While Cui et al. (2021) and Wang et al. (2021) reported higher abundance of *Bacteroidetes* in males, England et al. (2024) found the opposite, even though males exhibited higher BWG in all three studies. A consistent finding, however, is that male associated-microbiota supports better protein and fatty acid metabolism, whereas female microbiota tends to harbor more species associated with mucin degradation in the gut (Cui et al., 2021; England et al., 2024). These differences may contribute to sex-based variations in nutrient utilization and growth. Additionally, male birds typically possess larger lungs, which has been linked to higher metabolic activity (Shalev and Pasternak, 1998). Consistent with previous findings, female birds in this study achieved lower final body weights while depositing more fat (Benyi et al., 2015; England et al., 2022; Maynard et al., 2023; Shalev and Pasternak, 1998). The higher fat deposition observed in female broilers may not solely result from their lower ME requirements, where excess energy is stored as fat. Rather, it likely reflects inherent physiological differences between sexes, with increased fat deposition in females serving as a preparatory adaptation for the reproductive stage. Interestingly, the energy requirement for muscle development appears to be similar between sexes (Shalev and Pasternak, 1998). Among the nutrient digestibility measured in the current study, starch was the only one equally digested between both sexes. Starch, a primary energy source, is digested by amylase enzymes into sugars (Aderibigbe et al., 2020). Broilers have demonstrated a high capacity for starch digestion, even shortly after hatching (Svihus, 2014). Unlike protein and fat digestion, which involve multiple complex pathways and are heavily influenced by hormones, starch digestion is relatively simple, potentially explaining the lack of sex or genotype differences in this parameter (Le Bihan-Duval et al., 1998).

Carcass yield analysis revealed significant sex-related differences. Female birds exhibiting lower breast muscle yield and higher fat pad percentages than males. While the general consensus in literature holds that male broilers grow to heavier BW, resulting in higher absolute values for breast and leg cuts, there are inconsistencies when yield is expressed as a percentage of BW. Some studies report higher breast yields in females despite their lower body weights (Goo et al., 2019; Maynard et al., 2023; Wang et al., 2021). In our study, males exhibited a significantly higher total breast meat yield compared to females, which corresponds with the higher protein digestibility observed in male birds. As previously discussed, hormonal influences play a substantial role in fat deposition in female birds (Le Bihan-Duval et al., 1998). Combined with their lower energy requirements, this likely contributes to the higher fat percentages observed in females in the current study. Benyi et al. (2015) noted that female birds begin depositing fat around two weeks earlier than males. These findings suggest that female broilers reach peak BW earlier, after which part of the ingested energy is diverted towards fat deposition in preparation for sexual maturity, rather than being utilized for lean muscle development (Benyi et al., 2015; England et al., 2023; Maynard et al., 2023; Nogueira et al., 2019).

Despite significant differences in overall body weight between Cobb and Ross broilers, breast muscle yield was similar across the two genotypes. According to performance objectives provided by both Cobb-Vantress and Aviagen, breast yield typically increases proportionally with body weight (Aviagen, 2022b; Cobb Vantress, 2022). However, in the present study, the higher body weights observed in Cobb birds did not translate into greater breast muscle yield compared to Ross birds. This finding aligns with the higher overall (0–42 d) digestible lysine (balanced protein) intake, yet lower conversion efficiency per kilogram of body weight in Cobb, suggesting that Ross birds are more efficient at converting ingested protein into muscle tissue. This interpretation is further supported by the numerically lower crude protein digestibility observed in Cobb birds. Meanwhile, the higher body weight in Cobb was associated with significantly greater fat pad deposition compared to Ross, which is consistent with the higher ME conversion observed in Cobb birds.

From an economic perspective, the 5–6 % higher body weight in Cobb resulted in approximately 9 % greater feed cost per bird and only about 6 % more carcass output, indicating that a carcass-to-feed price ratio of approximately 1.08–1.10 would be required to achieve a positive return under current feed cost conditions.

The rapid broiler growth rate has been associated with a rise in breast muscle myopathies, including woody breast and white striping (Bordignon et al., 2022; Kuttappan et al., 2012; Trocino et al., 2015). Although their external manifestations are well documented, the underlying causes of these myopathies are still largely unclear (Toghyani et al., 2025). White striping has been strongly linked to accelerated growth rates (Kuttappan et al., 2012), which is consistent with our observations in the current study, where Cobb broilers, which grew quicker than Ross, exhibited nearly twice the average white striping score. This trend was also evident when comparing male and female birds (Bordignon et al., 2022; Trocino et al., 2015). Aligning with previous research female birds in the current study, with a slower growth rate and having lower BW, had significantly lower occurrence and intensity of white striping and woody breast compared to male birds (Kuttappan et al., 2012; Trocino et al., 2015). These findings support previous research linking accelerated growth rates to increased incidences of meat quality defects (Bordignon et al., 2022; Toghyani et al., 2025).

## Conclusion

Results from the current study indicate that irrespective of dietary nutrient density, Cobb birds exhibited faster growth rate, achieving higher body weights than Ross by day 42. However, evidence of compensatory growth was observed in Ross birds during the final week suggesting that if the experimental period had been extended beyond 42 days, Ross birds may have achieved comparable BW to Cobb. While dietary nutrient density did not significantly impact overall BWG, Cobb specs resulted in increased FI and correspondingly higher FCR. However, this higher FI did not result in significant differences in nutrient digestibility between the two genotypes. Nonetheless, aligned with higher FCR, the increased nutrient intake resulted in higher calculated energy and digestible lysine conversion to body weight in Cobb birds.

Additionally, the results support the potential for reducing dietary energy density for younger broilers (< 24 days) without compromising growth performance, offering a practical strategy to improve cost effectiveness in broiler production. The superior feed efficiency observed in Cobb birds fed either Ross or AGF specifications suggest that both genotypes respond favorably to increased amino acid density, even when dietary energy is reduced during earlier growth phases, as seen in the AGF specifications.

Sex significantly influenced both BWG and nutrient digestibility. Female birds exhibited lower BW, higher fat deposition, and reduced digestibility of protein, energy, and fat compared to males. This reduced growth rate was associated with a lower incidence of breast myopathies. These findings suggest that further investigation into sex-related differences in metabolism and gut microbiota may facilitate the development of more targeted nutritional and management strategies to optimize broiler performance.

## CRediT authorship contribution statement

**Carria Xie:** Writing – original draft, Investigation, Formal analysis, Data curation. **Shemil Macelline:** Writing – review & editing, Formal analysis, Data curation. **Sonia Liu:** Writing – review & editing, Methodology, Funding acquisition, Conceptualization. **Mehdi Toghyani:** Writing – review & editing, Supervision, Project administration, Funding acquisition, Formal analysis.

## Disclosures

We declare that we have no financial and personal relationships with other people or organizations that can inappropriately influence our work, and there is no professional or other personal interest of any nature or kind in any product, service and/or company that could be construed as influencing the content of this paper. The authors declare no conflicts of interest.

## References

[bib0001] Aderibigbe A., Cowieson A., Sorbara J.O., Adeola O. (2020). Intestinal starch and energy digestibility in broiler chickens fed diets supplemented with α-amylase. Poult. Sci..

[bib0002] Aftab U. (2019). Energy and amino acid requirements of broiler chickens: keeping pace with the genetic progress. Worlds Poult. Sci. J..

[bib0003] Aviagen (2022).

[bib0004] Aviagen (2022).

[bib0005] Benyi K., Tshilate T.S., Netshipale A.J., Mahlako K.T. (2015). Effects of genotype and sex on the growth performance and carcass characteristics of broiler chickens. Trop. Anim. Health Prod..

[bib0006] Birk R.Z., Rubio-Aliaga I., Boekschoten M.V., Danino H., Müller M., Daniel H. (2014). Differential regulation of pancreatic digestive enzymes during chronic high-fat diet-induced obesity in C57BL/6J mice. Br. J. Nutr..

[bib0007] Bordignon F., Xiccato G., Boskovic Cabrol M., Birolo M., Trocino A. (2022). Factors affecting breast myopathies in broiler chickens and quality of defective meat: a meta-analysis. Front. Physiol..

[bib0008] Çapar Akyüz H., Onbaşılar E.E., Bayraktaroğlu A.G., Ceylan A. (2022). Age and sex related changes in fattening performance, dermatitis, intestinal histomorphology, and serum IgG level of slow-and fast-growing broilers under the intensive system. Trop. Anim. Health. Prod..

[bib0009] Chrystal P.V., Moss A.F., Khoddami A., Naranjo V.D., Selle P.H., Liu S.Y. (2020). Impacts of reduced-crude protein diets on key parameters in male broiler chickens offered maize-based diets. Poult. Sci..

[bib0010] Clune S., Crossin E., Verghese K. (2017). Systematic review of greenhouse gas emissions for different fresh food categories. J. Clean. Prod..

[bib0011] Cobb vantress (2022).

[bib0012] Collin A., Malheiros R.D., Moraes V.M.B., Van As P., Darras V.M., Taouis M., Decuypere E., Buyse J. (2003). Effects of dietary macronutrient content on energy metabolism and uncoupling protein mRNA expression in broiler chickens. Br. J. Nutr..

[bib0013] Corring T. (1980). The adaptation of digestive enzymes to the diet: its physiological significance. Reprod. Nutr. Dev..

[bib0014] Cui L., Zhang X., Cheng R., Ansari A.R., Elokil A.A., Hu Y., Chen Y., Nafady A.A., Liu H. (2021). Sex differences in growth performance are related to cecal microbiota in chicken. Microb. Pathog..

[bib0015] Decuypere E., Onagbesan O., Swennen Q., Buyse J., Bruggeman V. (2007). The endocrine and metabolic interface of genotype-nutrition interactions in broilers and broiler breeders. Worlds Poult. Sci. J..

[bib0016] Dozier W.A., Gehring C.K. (2014). Growth performance of Hubbard × Cobb 500 and Ross × Ross 708 male broilers fed diets varying in apparent metabolizable energy from 14 to 28 days of age. J. Appl. Poult. Res..

[bib0017] Druyan S. (2010). The effects of genetic line (broilers vs. layers) on embryo development. Poult. Sci..

[bib0018] England A., Gharib-Naseri K., Kheravii S.K., Wu S.-B. (2023). Influence of sex and rearing method on performance and flock uniformity in broilers—Implications for research settings. Anim. Nutr..

[bib0019] England A.D., Gharib-Naseri K., Kheravii S.K., Wu S.-B. (2022). Rearing broilers as mixed or single-sex: relevance to performance, coefficient of variation, and flock uniformity. Poult. Sci..

[bib0020] England A.D., Heras-Saldana S.D.L., Gharib-Naseri K., Kheravii S.K., Wu S.-B. (2024). The effect of sex and dietary crude protein level on nutrient transporter gene expression and cecal microbiota populations in broiler chickens. Poult. Sci..

[bib0021] Goo D., Kim J.H., Choi H.S., Park G.H., Han G.P., Kil D.Y. (2019). Effect of stocking density and sex on growth performance, meat quality, and intestinal barrier function in broiler chickens. Poult. Sci..

[bib0022] Jørgensen H., Jakobsen K., Eggum B.O. (1993). Determination of endogenous fat and fatty acids at the terminal ileum and on faeces in growing pigs. Acta Agric. Scand. Sect. A Anim. Sci..

[bib0023] Kuttappan V.A., Brewer V.B., Apple J.K., Waldroup P.W., Owens C.M. (2012). Influence of growth rate on the occurrence of white striping in broiler breast fillets. Poult. Sci..

[bib0024] Kuttappan V.A., Hargis B.M., Owens C.M. (2016). White striping and woody breast myopathies in the modern poultry industry: a review. Poult. Sci..

[bib0025] Le Bihan-Duval E., Mignon-Grasteau S., Millet N., Beaumont C. (1998). Genetic analysis of a selection experiment on increased body weight and breast muscle weight as well as on limited abdominal fat weight. Br. Poult. Sci..

[bib0026] Maynard C.J., Maynard C.W., Jackson A.R., Kidd M.T., Rochell S.J., Owens C.M. (2023). Characterization of growth patterns and carcass characteristics of male and female broilers from four commercial strains fed high or low density diets. Poult. Sci..

[bib0027] Muñoz J.A., Suckeveris D., Demuner L.F., Caetano V.C., Silva A.D.L., Almeida T.W.D., Faria Filho D.E.D., Faria D.E.D. (2023). Productive and economic performance of broiler chickens fed diets with different nutritional levels. Anais da Acad. Brasil. Ciênc..

[bib0028] Nogueira B., Reis M., Carvalho A., Mendoza E., Oliveira B., Silva V., Bertechini A. (2019). Performance, growth curves and carcass yield of four strains of broiler chicken. Braz. J. Poult. Sci..

[bib0029] Pelletier N. (2008). Environmental performance in the US broiler poultry sector: life cycle energy use and greenhouse gas, ozone depleting, acidifying and eutrophying emissions. Agric. Syst..

[bib0030] Petit V., Arnould L., Martin P., Monnot M.-C., Pineau T., Besnard P., Niot I. (2007). Chronic high-fat diet affects intestinal fat absorption and postprandial triglyceride levels in the mouse. J. Lipid Res..

[bib0031] Pirgozliev V., Beccaccia A., Rose S.P., Bravo D. (2015). Partitioning of dietary energy of chickens fed maize-or wheat-based diets with and without a commercial blend of phytogenic feed additives. J. Anim. Sci..

[bib0032] Ravindran V., Dikeman M. (2024). Nutrition of Meat Animals: poultry. Pages 8-16 in Encyclopedia of Meat Sciences.

[bib0033] Ravindran V., Tancharoenrat P., Zaefarian F., Ravindran G. (2016). Fats in poultry nutrition: digestive physiology and factors influencing their utilisation. Anim. Feed Sci. Technol..

[bib0034] Ravindran V., Wu Y., Hendriks W. (2004). Effects of sex and dietary phosphorus level on the apparent metabolizable energy and nutrient digestibility in broiler chickens. Arch. Anim. Nutr..

[bib0035] Sakomura N.K., Longo F.A., Oviedo-Rondon E.O., Boa-Viagem C., Ferraudo A. (2005). Modeling energy utilization and growth parameter description for broiler chickens1. Poult. Sci..

[bib0036] Shalev B.A., Pasternak H. (1998). The relative energy requirement of male vs female broilers and turkeys. Poult. Sci..

[bib0037] Siriwan P., Bryden W.L., Mollah Y., Annison E.F. (1993). Measurement of endogenous amino acid losses in poultry. Br. Poult. Sci..

[bib0038] Snook J.T. (1971). Effect of diet on development of exocrine pancreas of the neonatal rat. Am. J. Physiol..

[bib0039] Stracke J., Volkmann N., May F., Döhring S., Kemper N., Spindler B. (2021). Walking on tiptoes: digital pads deserve increased attention when scoring footpad dermatitis as an animal welfare indicator in turkeys. Front. Vet. Sci..

[bib0040] Svihus B. (2011). The gizzard: function, influence of diet structure and effects on nutrient availability. Worlds Poult. Sci. J..

[bib0041] Svihus B. (2014). Starch digestion capacity of poultry1. Poult. Sci..

[bib0042] Tancharoenrat P., Ravindran V., Zaefarian F., Ravindran G. (2014). Digestion of fat and fatty acids along the gastrointestinal tract of broiler chickens. Poult. Sci..

[bib0043] Teeter R.G., Smith M.O. (1985). Feed intake effects upon gain, carcass yield, and ration digestibility in broilers force fed five Feed intakes. Poult. Sci..

[bib0044] Toghyani M., Macelline S., Selle P.H., Liu S.Y. (2024). Interactive effects of dietary energy levels with amino acid density on growth performance and optimal digestible lys to energy ratio of male broiler chickens. Poult. Sci..

[bib0045] Toghyani M., Macelline S., Selle P.H., Liu S.Y. (2025). Interactive effect of dietary metabolizable energy levels with amino acid density in male broiler chickens: carcass yield, nutrient intake, digestibility and excretion. Poult. Sci..

[bib0046] Tona K., Onagbesan O.M., Kamers B., Everaert N., Bruggeman V., Decuypere E. (2010). Comparison of Cobb and Ross strains in embryo physiology and chick juvenile growth. Poult. Sci..

[bib0047] Trocino A., Piccirillo A., Birolo M., Radaelli G., Bertotto D., Filiou E., Petracci M., Xiccato G. (2015). Effect of genotype, gender and feed restriction on growth, meat quality and the occurrence of white striping and wooden breast in broiler chickens. Poult. Sci..

[bib0048] Tumová E., Teimouri A. (2010). Fat deposition in the broiler chicken: a review. Sci. Agricult. Bohem..

[bib0049] Van Den Oever A.C.M., Bolhuis J.E., Van De Ven L.J.F., Kemp B., Rodenburg T.B. (2020). High levels of contact dermatitis and decreased mobility in broiler breeders, but neither have a relationship with floor eggs. Poult. Sci..

[bib0050] Van Der Eijk J.A.J., Van Harn J., Gunnink H., Melis S., Van Riel J.W., De Jong I.C. (2023). Fast- and slower-growing broilers respond similarly to a reduction in stocking density with regard to gait, hock burn, skin lesions, cleanliness, and performance. Poult. Sci..

[bib0051] Wang L.-D., Zhang Y., Kong L.-L., Wang Z.-X., Bai H., Jiang Y., Bi Y.-L., Chang G.-B., Chen G.-H. (2021). Effects of rearing system (floor vs. cage) and sex on performance, meat quality and enteric microorganism of yellow feather broilers. J. Intergr. Agric..

[bib0052] Wang M., Macelline S., Selle P.H., Liu S.Y., Toghyani M. (2025). Interactive effects of dietary grain and supplemental fat sources on growth performance and carcass characteristics of broiler chickens. Poult. Sci..

[bib0053] Xie C., Macelline S., Liu S., Toghyani M. (2024). Optimizing dietary energy and amino acid densities in male broiler chickens: impacts on performance, cost efficiency, and carcass quality. J. Appl. Poult. Res..

[bib0054] Yang Z., Pirgozliev V.R., Rose S.P., Woods S., Yang H.M., Wang Z.Y., Bedford M.R. (2020). Effect of age on the relationship between metabolizable energy and digestible energy for broiler chickens. Poult. Sci..

